# Role of L1CAM in retinoblastoma tumorigenesis: identification of novel therapeutic targets

**DOI:** 10.1002/1878-0261.13054

**Published:** 2021-07-18

**Authors:** Oliver Dräger, Klaus Metz, Maike Busch, Nicole Dünker

**Affiliations:** ^1^ Institute of Anatomy II Department of Neuroanatomy University of Duisburg‐Essen Medical Faculty Germany; ^2^ Institute of Pathology University of Duisburg‐Essen Medical Faculty Germany

**Keywords:** ADAM, CAM assay, chemoresistance, etoposide, L1, RB

## Abstract

The study presented focuses on the role of the neuronal cell adhesion molecule L1 cell adhesion molecule (L1CAM) in retinoblastoma (RB), the most common malignant intraocular childhood tumor. L1CAM is differentially expressed in a variety of human cancers and has been suggested as a promising therapeutic target. We likewise observed differential expression patterns for L1CAM in RB cell lines and patient samples. The two proteases involved in ectodomain shedding of L1CAM (L1CAM sheddases: ADAM10 and ADAM17) were likewise differentially expressed in the RB cell lines investigated, and an involvement in L1CAM processing in RB cells could be verified. We also identified ezrin, galectin‐3, and fibroblast growth factor basic as L1CAM signaling target genes in RB cells. Lentiviral *L1CAM* knockdown induced apoptosis and reduced cell viability, proliferation, growth, and colony formation capacity of RB cells, whereas *L1CAM‐*overexpressing RB cells displayed the opposite effects. Chicken chorioallantoic membrane assays revealed that *L1CAM* depletion decreases the tumorigenic and migration potential of RB cells *in vivo*. Moreover, *L1CAM* depletion decreased viability and tumor growth of etoposide‐resistant RB cell lines upon etoposide treatment *in vitro* and *in vivo*. Thus, L1CAM and its processing sheddases are potential novel targets for future therapeutic RB approaches.

## Graphical abstract

We investigated the role of the neuronal L1 cell adhesion molecule (L1CAM) in retinoblastoma (RB), a malignant intraocular childhood tumor. L1CAM depletion induced apoptosis and reduced RB cell viability *in vitro* and tumor growth and migration *in vivo*. Thus, L1CAM together with its processing sheddases (ADAM10 and ADAM17) and identified downstream targets (ezrin, β‐galactoside‐binding protein galectin‐3, and fibroblast growth factor basic) are promising targets for novel therapeutic approaches.

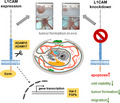

**Abbreviations**
ADAM: a disintegrin and metalloproteinase
CAM: chicken chorioallantoic membrane
FGFb: fibroblast growth factor basic
FGFR: fibroblast growth factor receptor
GAL‐3: β‐galactoside‐binding protein galectin‐3
L1CAM: L1 cell adhesion molecule
miRs: microRNAs
PMA: phorbol 12‐myristate 13‐acetate
RB: retinoblastoma

## 1 Introduction

The L1 cell adhesion molecule (L1CAM; CD171) is a 200–220 kDa transmembrane glycoprotein, which belongs to the immunoglobulin (Ig) superfamily of cell adhesion molecules. It is composed of six Ig‐like domains and five fibronectin type III repeats followed by a transmembrane region and a cytoplasmic tail [1, 2, 3, 4, 5]. L1CAM was originally described to play an essential role during brain development [6], where it is involved in migration processes and promotes neuron survival (for review, see Ref. [2, 3, 7]). Besides, L1CAM has been shown to play a pivotal role in the progression of human tumors [8, 9, 10]. It is believed that the ability of changing binding partners as well as its cleavage from the cell surface triggers L1CAM's functional switch from a static cell adhesion molecule to a motility promoting driver of cell migration in neural development but also in metastatic cancer progression [2, 5].

For paracrine signaling, constitutive and stimulated membrane cleavage of L1CAM is mediated by a disintegrin and metalloproteinases (ADAMs) and leads to the release of a soluble ectodomain termed L1–200 [2, 11, 12]. ADAMs are generally involved in induced and constitutive ectodomain shedding of several membrane‐bound proteins [13, 14, 15, 16, 17]. Release of L1CAM can be stimulated by shedding inducers like phorbol ester and pervanadate [11, 18]. The soluble ectodomain of L1CAM is functionally active and mediates cell migration [19], protection from apoptosis, and stimulation of cell survival [5, 20, 21, 22, 23]. Ectodomain shedding of L1CAM leads to a residual membrane‐bound 32 kDa C‐terminal fragment. Released 28 kDa L1CAM intracellular or cytoplasmic domain ultimately translocates into the nucleus, a discussed novel signaling mechanism and potential prerequisite for L1CAM mediated gene regulation [2, 5, 11, 24].

In addition, different microRNAs (miRs) have been implicated in the regulation of L1CAM. MiR‐146a and miR‐34a have been described as suppressors of L1CAM in gastric and endometrial carcinomas [25, 26], while miR‐29a and miR‐21‐3p directly or indirectly upregulate L1CAM [27, 28].

Retinoblastoma (RB) is the most common primary pediatric intraocular tumor [29]. Untreated, the tumor expands, may extend beyond the eye and develop metastatic spread [30]. Enucleation was the first successful RB therapy, but the implementation of new drug delivery routes, for example, intra‐arterial, intravitreal, or intracameral injections, significantly increased eye preservation rates and reduced systemic chemotherapy [31, 32]. Although chemotherapy is the mainstay of treatment to reduce tumor size, massive side effects and developing single drug or multidrug resistances against vincristine, etoposide or carboplatin, routinely used in combined RB VEC‐therapy, often limit treatment options as resistant tumor cells might eventually cause relapses [33]. Thirty‐five percent of RB patients develop secondary tumors and 50% of these patients die after therapy [34]. Therefore, developing new strategies to overcome drug resistances and reduce side effects by implementing molecularly targeted therapeutics with more effectiveness and less toxicity are major challenges for an optimization of RB management.

Adhesion molecules like L1CAM, which are involved in direct or indirect interactions between tumor cells and their microenvironment, have been identified as potential targets for treatment of cancers [35, 36], especially as L1CAM expression has been linked to augmented protection from apoptosis and increased chemoresistance in various cancer entities including RBs [20, 22, 37, 38]. Besides, a multitude of studies showed that L1CAM is overexpressed in a variety of human cancers (for review, see Ref. [8]) and in nearly all cancers L1CAM expression was associated with poor prognosis, tumor progression, and metastasis [8]. Accordingly, L1CAM has been proposed as a promising prognostic marker [4] as well as therapeutic target [4, 8, 39] (for review, see Ref. [35]) and the efficacy of L1CAM siRNA approaches has already been confirmed (for review, see Ref. [4, 8]).

In the study presented, we demonstrated the effect of L1CAM on RB cells' apoptosis, proliferation, viability, growth, and colony formation capacity *in vitro* as well as on their tumor formation and migration capacity *in vivo*. Besides, we verified the involvement of ADAM10 and ADAM17 as L1CAM processing sheddases in RB. Moreover, we identified ezrin, galectin‐3, and fibroblast growth factor basic (FGFb) as target genes of L1CAM signaling in RB. Finally, we could show that *L1CAM* depletion decreases viability and tumor growth of etoposide‐resistant RB cell lines upon etoposide treatment *in vitro* and *in vivo*.

## 2 Materials and methods

### 2.1 Human retina and retinoblastoma samples

Postmortem healthy human retina (hRet) and patient RB samples were used for comparative expression studies. The study methodologies conformed to the standards set by the Declaration of Helsinki. The Ethics Committee of the Medical Faculty of the University of Duisburg‐Essen approved the use of human retina (approval # 06‐30214) and RB samples (approval # 14‐5836‐BO) for research conducted in the course of the study presented, and written informed consent has been obtained from patients' relatives or parents.

### 2.2 Cell lines and culture

The human RB cell lines RB355, Rbl30, and RB247 [40], formerly donated by K. Heise, were kindly provided by Dr. H. Stephan. The RB cell lines Y79 [41] and WERI‐Rb1 [42], originally purchased from the Leibniz Institute DSMZ (German Collection of Microorganisms and Cell Cultures), were likewise kindly provided by Dr. H. Stephan. The cell lines were cultivated as suspension cultures as described previously [43]. Human embryonic kidney cells were grown as adherent cell culture in (Dulbecco's Modified Eagle Medium (DMEM; PAN‐Biotech, Aidenbach, Germany) with 10% FCS, 4 mm l‐glutamine, 100 U penicillin·mL^−1^, and 100 µg streptomycin·mL^−1^ at 37 °C, 5% CO_2,_ and 95% humidity. The corresponding etoposide‐resistant RB cell lines RB355_Etop, Y79_Etop, and WERI‐Rb1_Etop were established and kindly provided by Dr H. Stephan. The cultivation of the etoposide‐resistant RB cell lines was described previously by our group [44].

### 2.3 Plasmids and lentiviral expression vectors

To generate the L1CAM overexpression vector (pLenti_CMV_L1CAM), the human L1CAM cDNA sequence was cut from the phL1A‐pcDNA3 plasmid (#12307; Addgene, Watertown, MA, USA, [45]) via *Eco*RI fast digest restriction enzyme (Thermo Scientific, Oberhausen, Germany) and ligated into the *Eco*RI digested pENTR4 vector (#17424; Addgene, [46]). Full‐length L1CAM sequence was finally cloned into the pLENTI CMV Puro Dest vector (#17452; Addgene, [46]) by Gateway LR Clonase II Enzyme Mix (Invitrogen, Darmstadt, Germany), according to the manufacturer's protocol. The empty pLENTI_CMV Puro Dest vector was used as a control vector in all L1 overexpression experiments.

L1CAM knockdown was mediated by ‘Mission shRNA Plasmid DNA’ using shL1#668 (TRCN0000303668; Sigma‐Aldrich, München, Germany) and shL1#914 (TRCN0000063914; Sigma‐Aldrich) clones with a pLKO.2‐puro backbone. The shRNA control *pPRIME‐CMV‐Neo‐FF3* (p234; #11665; Addgene, [47]) containing a targeting hairpin sequence against firefly luciferase was used as a control in all transduction experiments. All vector constructs were verified by Sanger sequence analyses.

MicroRNA‐346 sequences were derived from genomic HEK293T DNA by RT‐PCR and specific primers [5′‐CGGAATTCGAATTTGGCTGCAGGTTGGA‐3′ (forward) and 5′‐CGGGATCCGCTGACTGTGGAGGTAGGTT‐3′ (reverse)], containing *Eco*RI and *Bam*HI restriction sites (underlined). For miR‐346 binding studies, the miR‐346 binding site of the 3´‐UTR of the *L1CAM* sequence was amplified by RT‐PCR from genomic HEK293T DNA with specific primers [5′‐CGACTAGTCTGTTTTGCCAGCCCATTTG‐3 (forward); 5′‐CGGAGCTC TGGAGCAGAGATGGCAAAGA‐3′ (reverse)], containing *Spe*I and *Sac*I restriction sites (underlined). Afterward, RT‐PCR fragments were ligated into a *pCR*®*4‐TOPO* vector with the *TOPO*™*TA Cloning*™ *Kit* (Thermo Scientific) following the protocol of the manufacturers. After digestion with *Eco*RI/*Bam*HI (Thermo Scientific), the miR‐346 PCR products were ligated into a *pSG5* vector (#216201; Stratagene, La Jolla, CA, USA). After digestion with *Spe*I/*Sac*I (Thermo Scientific), the miR‐346 binding site PCR products were ligated into a pMIR‐TK‐RNL [48]. All vector constructs were verified by Sanger sequence analyses.

### 2.4 Luciferase assay

MiR‐346 binding to the 3′‐UTR of L1CAM was measured with the ‘Dual‐Luciferase® Reporter Assay System’ (Promega, Mannheim, Germany). HEK293T cells were transiently cotransfected with the pSG5‐miR‐ 346 or the control vector (pSG5) in combination with the pMIR‐TK‐RNL vector including wild‐type L1 binding sites. Additional cotransfection of pMIR‐TK‐RNL and pSG5 was carried out for normalization. After 48 h, cells were lysed in 1× Passive‐Lysis Buffer (Promega) and the luciferase activity was measured with the ‘Dual‐Luciferase reporter assay’ (E1910; Promega and Glomax 20/20 Luminometer) as described by the manufacturer. The relative luciferase activity was determined as the quotient of firefly luciferase and Renilla luciferase activity. Analyses were performed in triplicates.

### 2.5 L1CAM and miR‐146a‐5p overexpression in retinoblastoma cells

For transient L1CAM overexpression, 4 × 10^5^ Rbl30 or 3 × 10^5^ RB247 cells were seeded in six‐well plates in 2 mL DMEM (PAN‐Biotech) without penicillin/streptavidin. 4 µg of plasmid DNA (pLenti_L1CAM or empty pLenti_CMV_PuroDest as control) and 20 µL transfection reagent (FuGENE® HD; Promega, Walldorf, Germany) were mixed 1 : 5 in DMEM without supplements as previously described by our group [49]. For miR‐146a‐5p overexpression experiments, we seeded 5 × 10^5^ RB355, WERI‐Rb1, or Y79 cells in 2 mL of DMEM without penicillin/streptavidin and 4 µg of pcDNA3‐miR‐146a plasmid (Addgene, #15092 [50]) or pcDNA3.1 (Thermo Fisher Scientific) as negative control and 16 µL of transfection reagent as described above.

### 2.6 Stable L1CAM knockdown

For virus production, HEK293T cells were transfected as described previously [47] with each of the following plasmid DNAs: packaging vectors pczVSV‐G [51], pCD NL‐BH [51] and either pLENTI_CMV_L1CAM, pLENTI CMV Puro Dest vector (negative control for overexpression experiments), pLKO.2‐puro_shL1#668, pLKO.2‐puro_shL1#914 or pPRIME‐CMV‐Neo‐FF3 (p234; negative control for knockdown experiments).

Experimental conditions for the lentiviral transductions were the same as described previously [49]. Cells transduced with the shL1#668 knockdown clone had to be selected by adding 0.3 µg Puromycin·mL^−1^ (Invitrogen) to the cultivation medium for 5 days prior to knockdown analysis.

### 2.7 RNA extraction and quantitative real‐time PCR

RNA isolations from RB cells were performed using the NucleoSpin® RNA II Kit (Macherey & Nagel, Düren, Germany) and the miRNeasy Kit (Qiagen, Hilden, Germany), respectively.

For quantitative real‐time PCR analyses, cDNA was synthesized with the QuantiTect Reverse Transcription Kit (Qiagen) following the manufacturer's protocol and the following human TaqMan™ Gene Expression Assays (Applied Biosystems, Darmstadt, Germany) were used: *L1CAM* (Hs0060855_m1), hGAPDH (Hs99999905_m1), and *18S* (Hs99999901_s1). The latter was used as an endogenous control. In RT‐qPCRs, conducted in duplicates, 20 µL of a TaqMan Universal PCR Master Mix (Applied Biosystems) was used and the samples were run in a 7300 Real‐Time PCR System (Applied Biosystems) using the following program: 50 °C for 2 min, 95 °C for 10 min, 95 °C for 15 s, 60 °C for 60 s and 40 cycles.

For analysis of *ADAM10*, *ADAM17, Ezrin, Galecin‐3,* and *FGF‐basic* expression, a SYBR™ Green PCR assay (Applied Biosystems) was used with specific primers 5′‐CACGAGAAGCTGTGATTGCC‐3′ (forward) and 5′‐TCCGGAGAAGTCTGTGGTCT‐3′ (reverse) for whole *ADAM10*, 5′‐AGGATGCTTGGGATGTGAAGA‐3′ (forward) and 5′‐GTGAAAAGGTGTGCCAAGCA‐3′ (reverse) for whole *ADAM17,* 5′‐TGAGGAGAAGCGCATCACTG‐3′ (forward) and 5′‐TTATTCTCATCTCGGGCCTGG‐3′ (reverse) for whole ezrin, 5′‐TCTTCTGGACAGCCAAGTGC‐3′ (forward) and 5′‐TGTTATCAGCATGCGAGGCA‐3′ (reverse) for whole galectin‐3, 5′‐CCGTTACCTGGCTATGAAGG‐3′ (forward) and 5′‐AAAGAA ACACTCATCCGTAACACA‐3′ (reverse) for whole FGFb, and 5′‐ACCCACTCCTCCACCTTTGA‐3′ (forward) and 5′‐CTGTTGCTGTAGCCAAATTCGT‐3′ (reverse) for human *GAPDH* (h*GAPDH*) as an endogenous control.

RT‐PCRs were conducted in triplicates in 20 µL of SYBR^TM^ Green PCR Master Mix (Applied Biosystems) using the following program: 95 °C for 15 min; 94 °C for 15 s, 55 °C for 30 s and 70 °C for 34 s and 40 cycles.

For miRNA expression analyses, a miScript PCR Starter Kit (# 2181193; Qiagen) was used, following the instructions of the manufacturer. For the quantification of mature miRNAs, a designated miScript HiSpec Buffer (Qiagen) was used together with specific primers for hsa‐miR‐21‐3p (CAACACCAGTCGATGGGCTGT), hsa‐miR‐29a‐3p (TAGCACCATCTGAAATCGGTTA), hsa‐miR‐34a (5′‐ TGGCAGTGTCTTAGGTGGTTGT‐3′), hsa‐miR‐146a‐5p (TGAGAACTGAATTCCATGGGTT), hsa‐miR‐346 (TGTCTGCCCG CATGCCTGCCTCT), and *5.8S* RNA (5′‐CTACGCCTGTCT GAGCGTCGCTT‐3′) as an endogenous control. The reactions were performed in duplicates using the following program: 95 °C for 15 min; 94 °C for 15 s, 55 °C for 30 s and 70 °C for 34 s and 40 cycles.

RNA isolation from chicken chorioallantoic membrane (CAM) tissue punches was performed as described previously [43]]. Quantitative real‐time PCR analyses were performed and quantified following the protocol published previously [43].

### 2.8 Western blotting

For western blot analyses, PBS‐washed cells were lysed in RIPA buffer plus supplements [52] for 60 min at 4 °C on a shaker and afterward centrifuged at 10 000 ***g*** at 4 °C for 30 min. Protein concentration was measured by BCA assay (Thermo Scientific) according to the manufacturer's protocol. Equal amounts of protein extracts were separated on a 10–12% SDS/PAGE and transferred onto nitrocellulose membranes. Membranes were incubated with primary antibodies against L1 ectodomain (1 : 11 000; L4343‐25ul; Sigma‐Aldrich), L1 (1 : 1000; ab24345; Abcam, Cambridge, MA, USA), ADAM10 (1 : 1000; #14194; Cell Signaling, Danvers, Ma, USA), ADAM17 (1 : 1000; ab6326; Abcam, Cambridge, UK), Ezrin (1 : 1000; sc‐58758; Santa‐Cruz, Dallas, TX, USA), galectin‐3 (1 : 1000; #12733; Abcam), FGFb (1 : 1000; EPR20145‐227; Abcam), and β‐actin (1 : 1000; #4967; Cell Signaling) at 4 °C overnight. Species‐specific HRP‐conjugated secondary antibodies (goat anti‐rabbit; P0448; DAKO and rabbit anti‐mouse; P0260; DAKO, Glostrup, Denmark) were used in dilutions of 1 : 10 000 at room temperature for 1 h. The HRP signal was detected by adding Western Bright Chemiluminescence Reagent (Advansta, San Jose, CA, USA).

### 2.9 Cell viability assays

To determine cell viability, 4 × 10^4^ cells in 100 µL medium were seeded in a 96‐well plate in three duplicates. After 48 h of incubation, 10 µL of a water‐soluble tetrazolium (WST‐1) solution (Sigma‐Aldrich) was added to each well and cells were incubated at 37 °C for a designated period. The formazan product of viable cells was quantified in a microplate reader at an absorbance of 450 nm.

### 2.10 Growth kinetic

For growth kinetics analyses in a 24‐well plate format, 3 × 10^5^ cells were seeded in 500 µL supplemented DMEM in triplicates and the number of vital cells was determined by manual cell counts every 24 h (five time points: 0, 24, 48, 72, and 96 h) after trypan blue exclusion.

### 2.11 Cell proliferation and apoptosis detection

To determine cell proliferation, 4 h prior to PFA fixation 5 µm BrdU (5‐Bromo‐2′‐deoxyuridine; BrdU; Sigma‐Aldrich) was added to the cells. The BrdU signal was revealed by a rat anti‐BrdU primary antibody (1 : 1000; ab6326; Abcam, Cambridge, UK) and visualized by a Alexa Flour 594‐labeled goat anti‐rat secondary antibody (1 : 1000; Molecular Probes, Eugene, OR, USA). Changes in cell death levels were determined by manual counts of pyknotic nuclei after 4′,6‐Diamidino‐2‐phenylindole (DAPI; Sigma‐Aldrich) stains.

For each experiment, six coverslips were stained and the percentages of proliferating or apoptotic cells were calculated as described previously by our group [43, 53].

### 2.12 Soft agarose assay

Soft agarose assays were performed as described previously [43]. 5000 cells were seeded in 2 mL soft agarose in a six‐well dish in triplicates and cultivated for 3 weeks. Colony formation capacity (%) was calculated by counting the number of colony‐forming cells and viable single cells in six visual fields (10× magnification) in triplicates per assay. Colony size was measured by capturing images using a Nikon Eclipse TS2 microscope equipped with a digital camera and ic measure 1.0 software (Nikon, Düsseldorf, Germany). For the determination of colony size, eight colonies per well were measured.

### 2.13 Treatment with chemotherapeutics

To investigate chemosensitivity, L1CAM‐depleted RB cells were treated with different concentrations of etoposide (RB355: 1 µm, WERI‐Rb1: 5 µm, Y79: 3 µm). Seventy‐two hours upon treatment, cell viability was analyzed by cell viability WST‐1 assays.

### 2.14 ADAM10 and ADAM17 inhibitor studies

For ADAM10 and ADAM17 inhibitor studies, 5.0 × 10^5^ cells were seeded in 1 mL of DMEM without supplements per well of a 24‐well plate dish. Cells were treated with indicated concentrations of phorbol 12‐myristate 13‐acetate (PMA; Sigma‐Aldrich, Steinheim, Germany) diluted in equal volumes of DMSO (Sigma‐Aldrich) 30 min prior to treatment with 2 µm of ADAM10 inhibitor GI254023X (Sigma‐Aldrich) or 5 µm of ADAM17 inhibitor TAPI‐1 (Tocris, Minneapolis, MN, USA). DMSO‐treated cells served as negative controls. After 48 h of incubation, cell culture supernatant was collected and centrifuged at 10 000 ***g*** for 10 min at 4 °C. Equal volumes of cell‐free supernatant were analyzed with regard to soluble L1CAM by western blot analysis as described in previously.

### 2.15 CAM Assays

In order to test for changes in tumor formation and migration capacity, L1CAM‐depleted RB cells and control cells were grafted on the CAM mainly following Zijlstra and Palmers protocols [54, 55]. Twenty eggs were grafted in at least three independent experiments with 1 × 10^6^ cells. Seven days after grafting (E10–17), tumors which formed from the grafted cells were excised, measured, and photographed as described previously [44, 49, 56].

Intravenous injection of GFP‐labeled RB355 and WERI‐Rb1 control and L1CAM knockdown cells was carried out as described previously by our group [43]. Five days after injection, the chicken embryos were sacrificed and tissue punches (six per egg) of the ventral CAM opposing the injection site were collected and processed as described by Kim *et al*. [57] and Palmer *et al*. [54]. Successful injection of the GFP‐labeled cells was revealed by fluorescence microscopy of the CAM punches. Sample pools of at least three punches with detectable GFP‐labeled cells were used for further analysis. RNA isolations and quantification of hGAPDH in the CAM punches were performed as described previously [43].

For whole‐mount stainings, CAM punches were fixed in 4% PFA (Sigma‐Aldrich) overnight at 4 °C on a table top shaker in 24‐well plates. CAM punches were washed three times with Tris‐buffered saline (TBS; 150 mm NaCl; Carl‐Roth, Karlsruhe, Germany; 20 mm Tris/HCl; Carl‐Roth) supplemented with 0.1% Triton X‐100 (Sigma‐Aldrich) for 30 min at room temperature on a shaker. Blocking was carried out by incubation with 1× PBS with 3% BSA (Carl‐Roth) for 1 h while shaking. The specific first antibody against chicken desmin (D33; ab8470, Abcam, Cambridge, UK) was diluted 1 : 20 in 1× PBS with 3% BSA and incubated overnight in a humidified chamber at 4 °C. Tissue samples were washed in 1×PBS with 1% Triton X‐100 for 10 min and three times with 1×PBS with Triton X‐100 and 20% BSA for 1 h on a shaker at room temperature. Alexa Fluor®594 goat anti‐mouse IgG (Molecular Probes) was diluted 1 : 1000 in 1×PBS, and CAM punches were incubated on a shaker over night at 4 °C. The next day, CAM samples were washed three times with 1xPBS with 1% Triton X‐100 at room temperature for 30 min on a shaker. Finally, samples were placed on slides and mounted with fluorescent mounting media (DAKO). Fluorescence microscopy was carried out with Nikon ECLIPSE E600 microscope and Nikon nis elements imaging 5.20.02 software (Nikon).

### 2.16 Statistical analysis

All assays were performed at least in triplicates. Statistical analyses were performed using graphpad prism 6 (Graphpad, San Diego, CA, USA). Data represent means ± SEM of three independent experiments from independent RB cell cultures. Results were analyzed by a Student's *t*‐test or one‐way ANOVA and Newman–Keuls post‐test and considered significantly different if *P*‐value < 0.05m (*), *P*‐value < 0.01 (**), or *P*‐value < 0.001 (***). Statistics on the growth curves was performed using a free web interface http://bioinf.wehi.edu.au/software/compareCurves/, which uses the compareGrowthCurves function from a Statistical Modeling package called statmod, available from the r Project for Statistical Computing: http://www.r‐project.org, previously described elsewhere [58].

## 3 Results

### 3.1 L1CAM is differentially expressed in retinoblastoma cell lines and RB patient samples

**Fig. 1:**
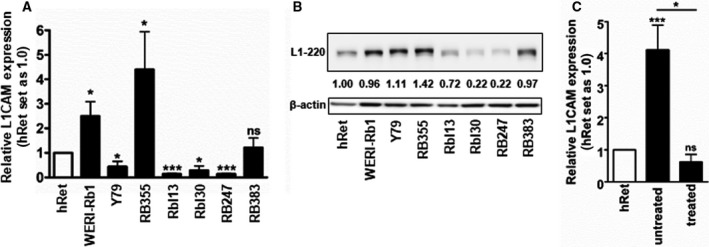
Endogenous L1CAM expression in RB cell lines and RB tumor specimens. Depiction of endogenous L1CAM expression in human retina and RB cell lines as revealed by quantitative real‐time PCR (A) and western blot (L1–220) (B) analyses. The indicated intensity ratios relative to ß‐actin, used as a loading control in (B), were calculated using micro manager 1.4 software (University of California, San Francisco, CA, USA). (C) L1CAM expression levels in enucleated RB patient eyes after treatment with chemotherapeutics (treated) and without prior treatment (untreated) in comparison with a hRet pool. A total of 16 RB tumor specimens were analyzed, 13 untreated and 3 treated specimens. Values are means of at least three independent experiments ± SEM. ns *P* > 0.05; *P < 0.05; and ***P < 0.001 statistical differences compared to the control group calculated by Student's *t*‐test or one‐way ANOVA with Newman–Keuls post‐test.

We analyzed the expression of L1CAM in the suspension RB cell lines WERI‐Rb1, Y79, Rbl13, Rbl30, RB247, and RB383 as well as in the adherent cell line RB355. Compared to the hRet, *L1CAM* was differentially expressed with significantly higher mRNA levels in WERI‐Rb1 and RB355 cells and significantly decreased expression in Y79, Rbl13, Rbl30, and RB247 cells (Fig. 1A). Western blot analysis mainly confirmed this expression pattern at L1CAM protein level (Fig. 1B).

In addition, we found a significant increase in L1CAM expression in RB patient tumors in comparison with the hRet (Fig. 1C). Compared to untreated specimen, a significant reduction in L1CAM expression was observed in the chemotherapy‐treated RB tumor samples investigated (Fig. 1C).

### 3.2 L1CAM knockdown induces apoptosis and reduces cell viability, proliferation, growth, and colony formation capacity of RB355 and WERI‐Rb1 cell lines

**Fig. 2:**
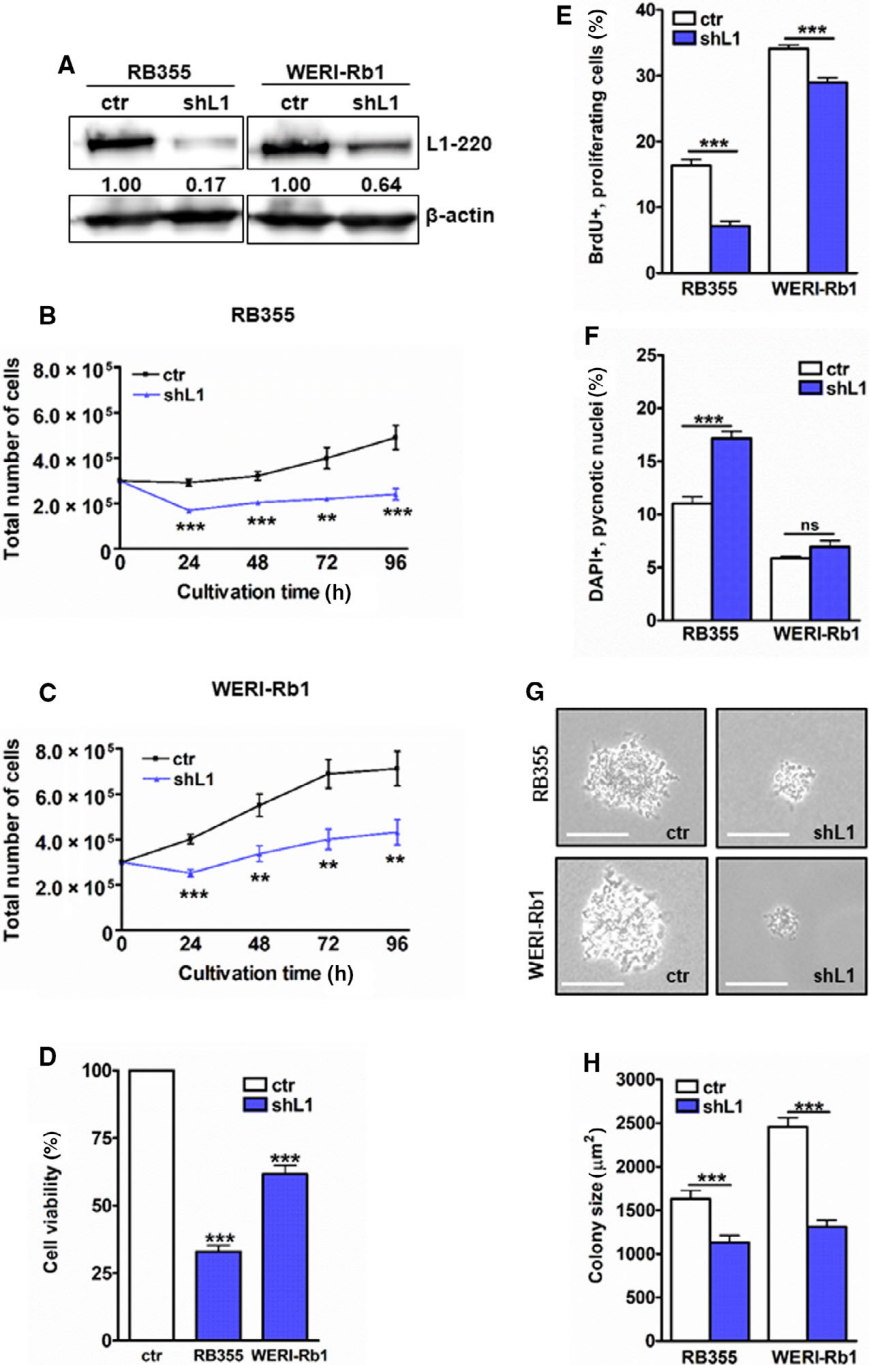
Effects of L1CAM knockdown on cell growth, apoptosis levels, and colony formation capacity of RB cells. (A) Verification of an efficient stable, lentiviral L1CAM knockdown (shL1) in RB355 and WERI‐Rb1 cells as revealed by western blot analysis. The indicated intensity ratios relative to ß‐actin, used as a loading control, were calculated using micro manager 1.4 software. Stable L1CAM knockdown significantly reduces cell growth of RB355 and WERI‐Rb1 cells reducing cell viability and proliferation levels compared to control cells (ctr) as revealed by growth curves (B, C), WST‐1 assays (D), and BrdU stains (E). L1CAM‐depleted RB355 RB cells show higher apoptosis levels as revealed by DAPI cell counts (F), and both RB cell lines show significantly reduced colony sizes as revealed by soft agarose assays (G, H). Values are means of three independent experiments ± SEM. ns *P* > 0.05; **P < 0.01; and ***P < 0.001 statistical differences compared to the control group calculated by Student's *t*‐test.

We performed *L1CAM* knockdown experiments in the RB cell lines RB355 and WERI‐Rb1, both exhibiting decent endogenous *L1CAM* levels. Testing different *L1CAM*‐specific shRNA clones, we achieved a very efficient knockdown as confirmed by quantitative real‐time PCR (Fig. S1A) and western blot analysis (Fig. 2A). Following *L1CAM* knockdown, both RB cell lines investigated exhibited a significantly lower cell viability and decreased growth as revealed by growth curve analyses (Fig. 2B,C), WST‐1 assays (Fig. 2D), and BrdU cell counts (Fig. 2E). L1CAM knockdown resulted in a significant increase in apoptosis levels of RB355 cells, while the apoptosis levels of WERI‐Rb1 cells did not significantly change (Fig. 2F). Besides, compared to their parental counterparts L1CAM‐depleted RB355 and WERI‐Rb1 cells formed significantly smaller colonies in soft agarose assays testing for changes in anchorage‐independent growth capability (Fig. 2G,H). These results confirmed the findings in other tumor entities, that a L1CAM knockdown leads to decreased tumor cell growth.

### 3.3 L1CAM knockdown decreases tumorigenicity and migration potential of RB cells *in vivo*

**Fig. 3:**
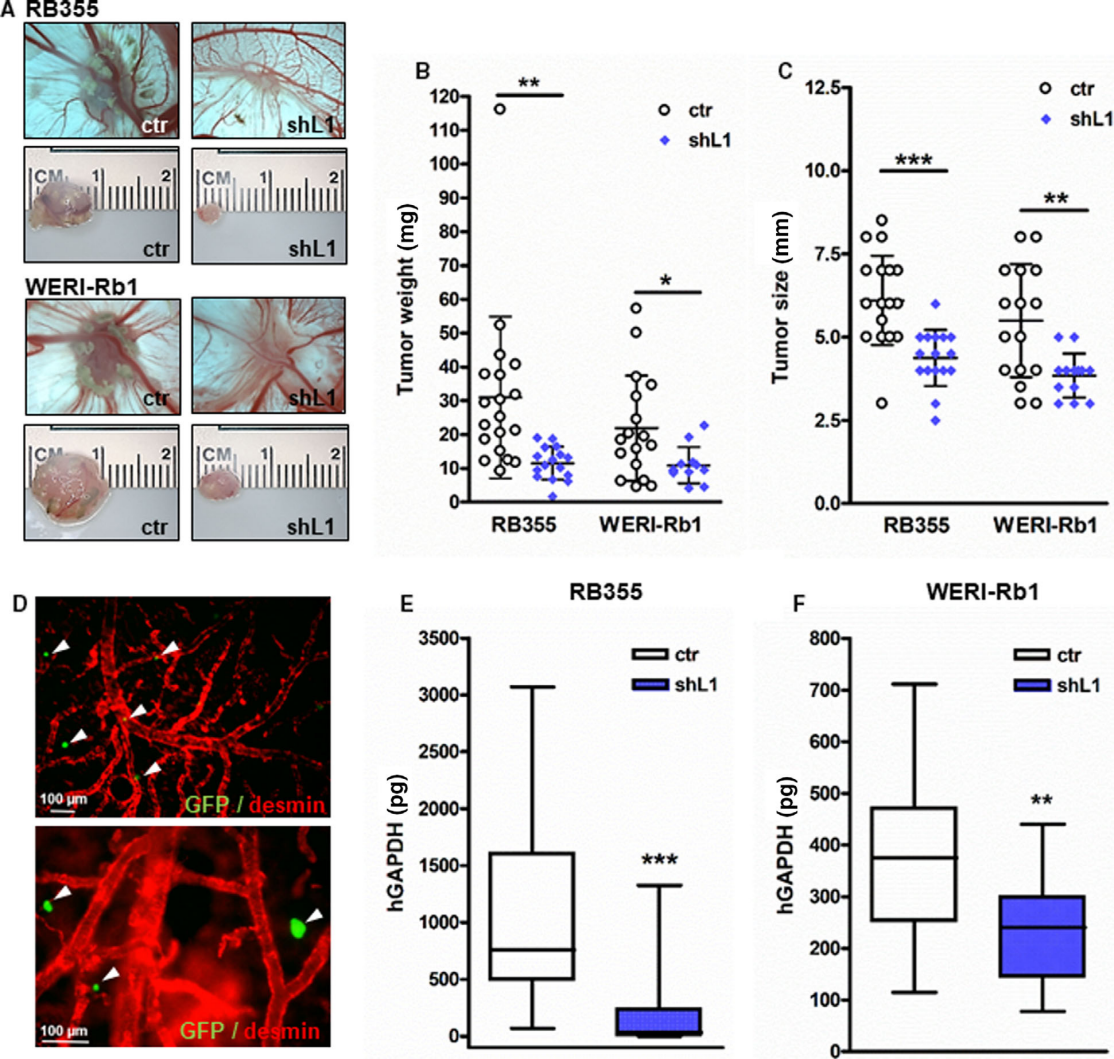
Effects of stable, lentiviral L1CAM knockdown on tumor formation and tumor cell migration of RB cells *in vivo*. (A) Photographs of CAM tumors *in situ* (upper row) and ruler measurements (in cm) of excised tumors (lower row) revealing that tumors forming on the upper CAM 7 days after grafting of L1CAM‐depleted (shL1) RB355 and WERI‐Rb1 cells were significantly smaller compared to those arising from control cells (ctr). (B, C) Quantification of CAM tumor weight (B) and size (C). (D) Chicken anti‐desmin antibody stains of CAM vessels (red) of representative CAM whole mounts showing extravasated GFP‐labeled RB355 cells (green, marked with arrowhead). Lower picture is a higher magnification close‐up (200×) of the upper one (100×), scale bar: 60 µm. (E, F) Quantification of hGAPDH content (normalized against 18S RNA) in lower CAM punches 6 days after intravenous injection of L1‐depleted (shL1) and control RB355_GFP and WERI‐Rb1_GFP cells (ctr). Values are means of three independent experiments ± SEM. *P < 0.05; **P < 0.01; and ***P < 0.001 statistical differences compared to the control group calculated by Student's *t*‐test.

To investigate whether L1CAM influences RB cells' tumor growth, we used the CAM assay as *in vivo* model. L1CAM‐depleted RB355 and WERI‐Rb1 cells and control cells were inoculated onto the CAM of 10‐day‐old chicken embryos. Photo‐documentation of CAM tumors developing from inoculated RB cells (Fig. 3A) and quantification of tumor weight (Fig. 3B) and size (Fig. 3C) revealed that L1CAM‐depleted RB cells develop significantly smaller tumors (Fig. 3A,C) than control cells, exhibiting lower weight and size (Fig. 3B,C). There were no significant changes in the number of developing tumors (data not shown).

After injection of GFP‐labeled RB355 and WERI‐Rb1 cells into the CAM vein, L1CAM‐depleted RB cells extravasated from the CAM vasculature (Fig. 3D), but displayed a significantly lower migration rate compared to their respective controls as revealed by human GAPDH real‐time PCR analyses of lower CAM punches (Fig. 3E,F). These results indicate that depleted L1CAM expression leads to decreased tumorigenicity and migration potential *in vivo*.

### 3.4 L1CAM overexpression induces cell growth, viability, and colony formation capacity and inhibits apoptosis in Rbl30 and RB247 retinoblastoma cell lines

**Fig. 4:**
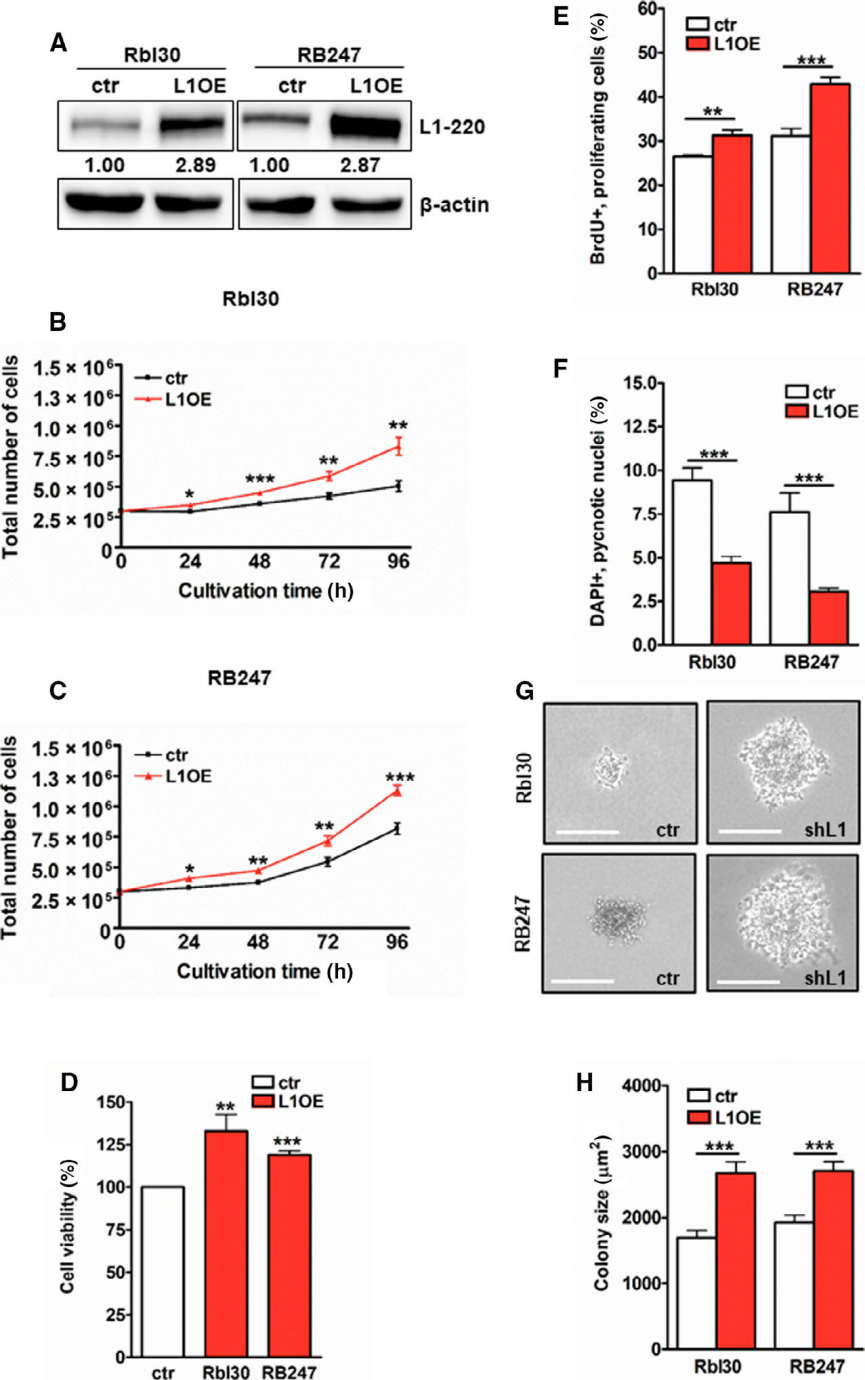
Effects of L1CAM overexpression on cell growth, apoptosis levels, and colony formation capacity of RB cell lines. (A) Transient L1CAM overexpression (L1OE) leads to a significant increase in L1CAM protein (L1–220) levels in Rbl30 and RB247 cells as revealed by western blot analysis. The indicated intensity ratios relative to ß‐actin, used as a loading control, were calculated using micro manager 1.4 software. (B, C) L1CAM overexpression results in significantly increased cell growth of Rbl30 and RB247 cells, accompanied by increased cell viability (D) and proliferation levels (E) compared to control cells (ctr). (F) L1CAM‐overexpressing Rbl30 and RB247 cells show significantly decreased apoptosis levels as revealed by DAPI cell counts. (G, H) As revealed by soft agarose assays, L1CAM overexpression results in increased colony sizes in both RB cell lines. Values are means of three independent experiments ± SEM. *P < 0.05; **P < 0.01; and ***P < 0.001 statistical differences compared to the control group calculated by Student's *t*‐test.

We performed L1CAM overexpression experiments in the RB cell lines Rbl30 and RB247, both exhibiting low endogenous L1CAM levels (Fig. 1A,B), to confirm L1CAM's effects on growth and apoptosis. Efficient L1CAM overexpression was verified by real‐time PCR (Fig. S1B) and confirmed by western blot analyses (Fig. 4A). *L1CAM*‐overexpressing RB cells exhibited significantly faster growth, higher cell viability, and increased proliferation as revealed by growth curve analyses (Fig. 4B,C), WST‐1 assays (Fig. 4D), and BrdU cell counts (Fig. 4E). Moreover, after L1CAM overexpression a significant decrease in apoptosis levels was detectable (Fig. 4F). Besides, anchorage‐independent colony formation capacity was significantly increased (data not shown) and compared to their parental counterparts L1CAM‐overexpressing Rbl30 and RB247 cells formed significantly larger colonies in soft agarose assays (Fig. 4G,H). The inverse effects seen compared to the L1CAM knockdown experiments confirm the protumorigenic potential of L1CAM overexpression in RB cell lines.

### 3.5 L1CAM knockdown lowers viability of etoposide‐resistant RB cell lines upon etoposide retreatment *in vitro*

**Fig. 5:**
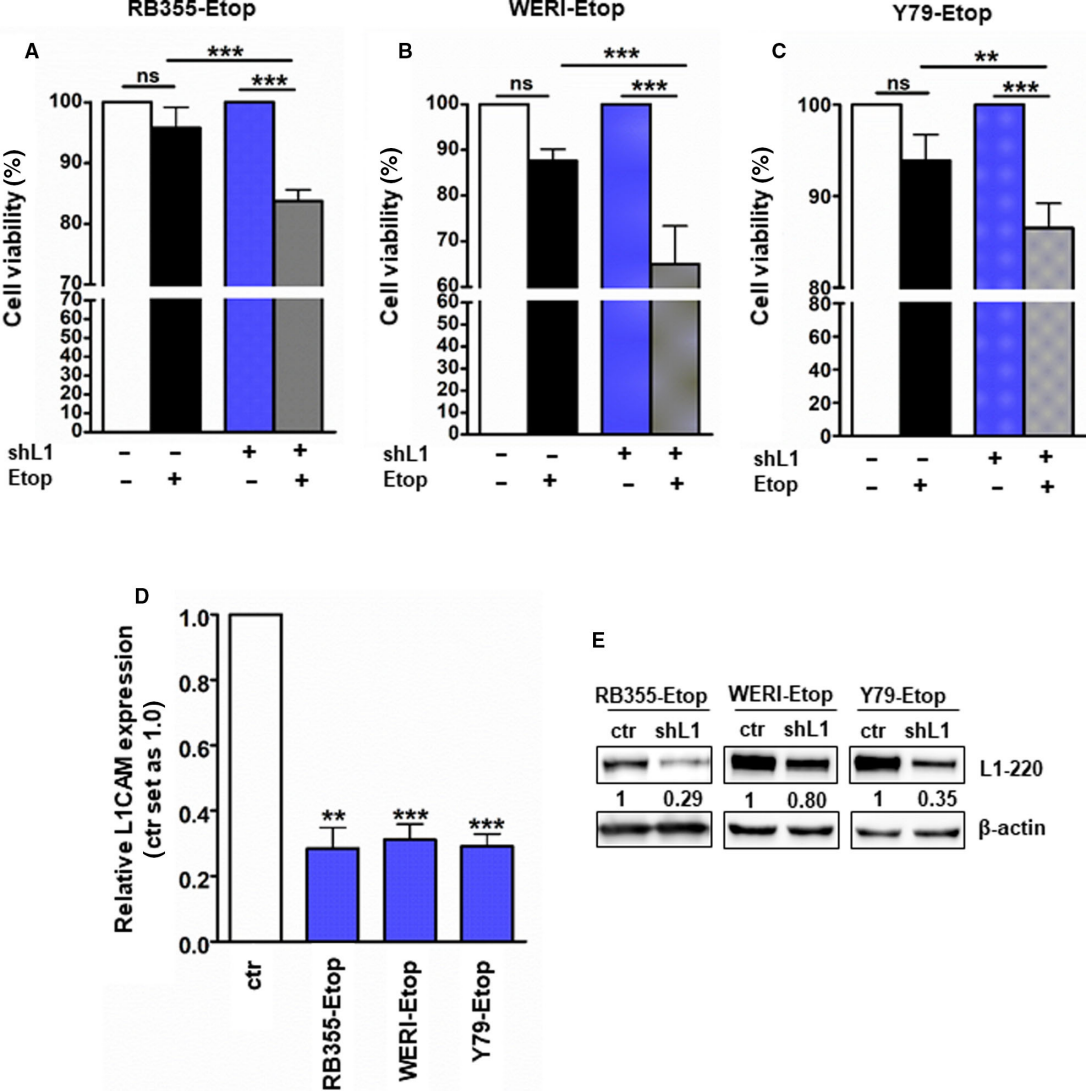
Effects of lentiviral L1CAM knockdown on viability of etoposide‐resistant RB cell lines. (A–C) WST‐1 assays showing that stable, lentiviral knockdown of L1CAM (shL1^+^) leads to significantly reduced cell viability of the etoposide‐resistant RB cell lines RB355‐Etop (A), WERI‐Rb1‐Etop (WERI‐Etop; B), and Y79‐Etop (C) simultaneously treated with indicated concentration of etoposide (Etop^+^). Cell viability was normalized to the respective untreated control group (Etop^−^). (D, E) Effective reduction of L1CAM expression levels upon lentiviral knockdown as revealed by quantitative real‐time PCR (D) and western blot analysis (E). The indicated intensity ratios relative to ß‐actin, used as a loading control, were calculated using micro manager 1.4 software. Values are means of three independent experiments ± SEM. ns *P* > 0.05; **P < 0.01; and ***P < 0.001 statistical differences compared to the control group calculated by paired Student's *t*‐test or one‐way ANOVA with Newman–Keuls post‐test.

We showed that L1CAM depletion in RB cells reduces cell viability, cell growth, and concomitantly induces apoptosis. Moreover, L1CAM was significantly downregulated in RB patients' tumors after chemotherapy (see above). Besides, in a previous study L1CAM knockdown in RB cells was associated with enhanced sensitivity against chemotherapeutics [38]. Thus, we set out to analyze the effect of stable, lentiviral L1CAM knockdown and etoposide retreatment on the viability of the etoposide‐resistant RB cell lines RB355, WERI‐Rb1, and Y79 (Fig. 5).

WST‐1 assays revealed that cell viability levels of all three etoposide‐resistant RB cell lines investigated significantly decreased after L1CAM depletion and retreatment with etoposide. While etoposide treatment of etoposide‐resistant RB355, WERI‐RB1, and Y79 control cells transduced with lentiviral control particles had no effect on the viability of these cell lines, treatment with etoposide significantly decreased cell viability of all L1CAM‐depleted etoposide‐resistant RB cell lines (Fig. 5A–C). Efficient L1CAM knockdown in the three etoposide‐resistant RB cell lines was verified by real‐time PCR (Fig. 5D) and confirmed by western blot analyses (Fig. 5E). These results indicate that L1CAM knockdown followed by etoposide retreatment lowers the viability of etoposide‐resistant RB cell lines *in vitro*.

### 3.6 L1CAM depletion decreases tumor growth of etoposide‐resistant RB cell lines upon etoposide treatment *in vivo*

**Fig. 6:**
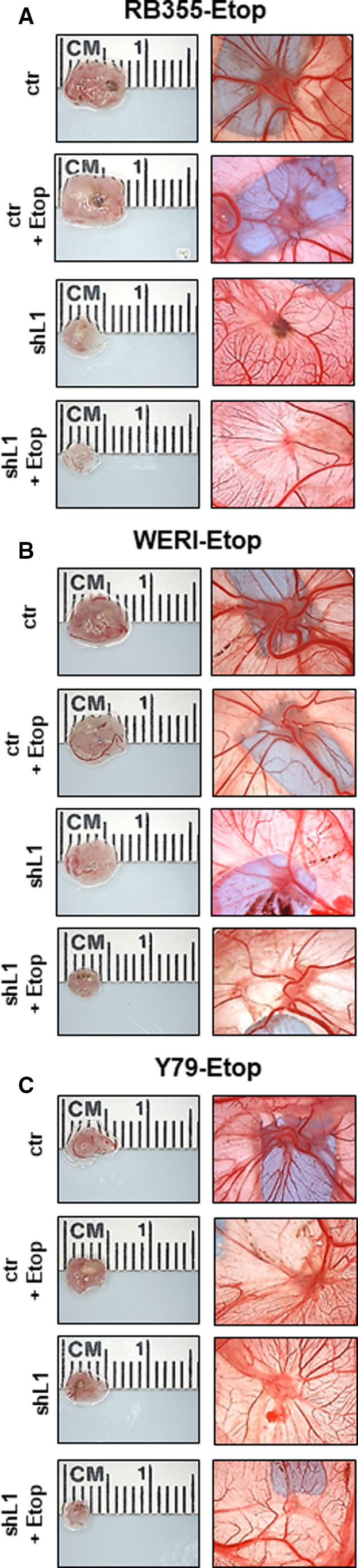
Effect of lentiviral L1CAM knockdown on CAM tumor formation of etoposide‐resistant RB cell lines upon etoposide treatment *in vivo*. (A–C) Photographs of ruler measurements (in cm) of excised CAM tumors (left column) and CAM tumors *in situ* (right column) 7 days after grafting the etoposide‐resistant RB cell lines RB355‐Etop (A), WERI‐Rb1‐Etop (WERI‐Etop, B), and Y79‐Etop (C). Compared to etoposide‐treated control cells (ctr), lentiviral *L1CAM* knockdown (shL1) results in the development of significantly smaller CAM tumors from all three etoposide‐resistant RB cell lines upon single etoposide treatment.

**Fig. 7:**
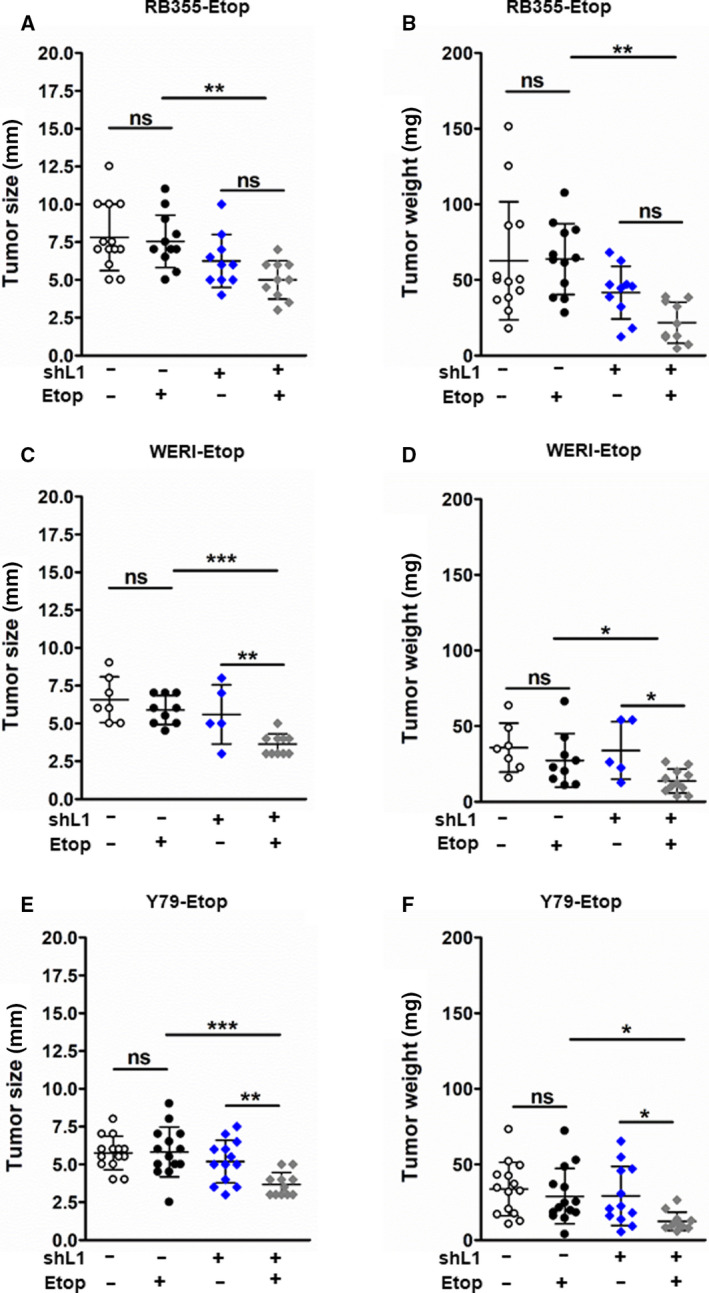
Effect of L1CAM knockdown on size and weight of tumors forming from etoposide‐resistant, drug‐treated RB cell lines *in vivo*. (A–F) Quantification of size and weight of CAM tumors developing from etoposide‐resistant, *L1CAM*‐depleted (shL1^+^) RB355‐Etop (A, B), WERI‐Rb1‐Etop (WERI‐Etop, C, D), and Y79‐Etop (E, F) cell lines treated once with etoposide (Etop^+^) or left untreated (Etop^−^). Values are means of four independent experiments ± SEM. ns *P* > 0.05; *P < 0.05; **P < 0.01; and ***P < 0.001 statistical differences compared to the control group calculated by one‐way ANOVA with Newman–Keuls post‐test.

To investigate whether L1CAM influences growth of chemoresistant RB tumors upon etoposide treatment, we again applied the CAM assay as an *in vivo* model. Etoposide‐resistant RB355, WERI‐Rb1, and Y79 cell lines with a stable, lentiviral *L1CAM* knockdown as well as control cells were inoculated onto the CAM of 10‐day‐old chicken embryos and treated once with etoposide. Photo‐documentation of CAM tumors developing from inoculated etoposide‐resistant RB355 cells (Fig. 6A), WERI‐Rb1 (Fig. 6B), and Y79 cells (Fig. 6C) and quantification of tumor weight (Fig. 7A,C,E) and tumor size (Fig. 7B,D,F) revealed that *L1CAM*‐depleted etoposide‐resistant RB cell lines develop significantly smaller tumors upon etoposide treatment than etoposide‐treated control cells.

### 3.7 ADAM10 and ADAM17 levels are increased in retinoblastoma cell lines and RB tumors and regulate L1CAM shedding

**Fig. 8:**
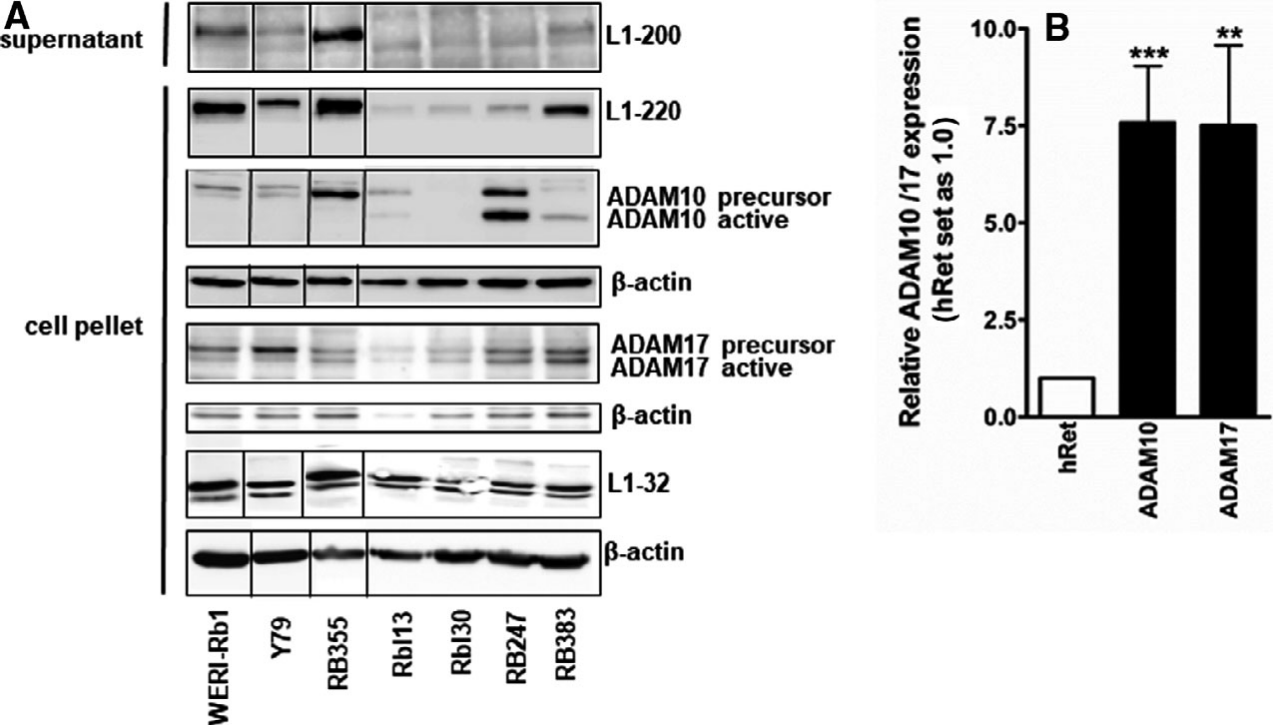
Endogenous L1CAM, ADAM10, and ADAM17 expression in RB cell lines as well as ADAM10 and ADAM17 expression in RB tumor specimens. (A) Western blot analysis of protein expression levels of L1CAM (L1–220), ADAM10, and ADAM17 in different RB cell lines and detection of the soluble 200 kDa L1 ectodomain (L1–200) as well as the C‐terminal fragment (L1–32) in cell culture supernatants and cell pellets. ß‐actin was used as a loading control. An endogenous cleavage of L1CAM and the presence of the soluble L1–200 ectodomain is clearly detectable in WERI‐Rb1, Y79, and RB355 cell culture supernatant. The RB cell lines analyzed show differential expression patterns for the precursor and active form of ADAM10 and ADAM17. (B) ADAM10 and 17 expression levels in enucleated RB patient eyes in comparison with a hRet pool. Values are means of 16 independent RB tumor specimens ± SEM. **P < 0.01 and ***P < 0.001 statistical differences compared to the control group calculated by Student's *t*‐test.

As ectodomain shedding of L1CAM is mediated by ADAM10 and ADAM17 and the soluble ectodomain of L1CAM is believed to mediate cell migration, protection from apoptosis, and stimulation of cell survival [2, 19, 20, 21, 22], we investigated the expression of these sheddases in seven RB cell lines by real‐time PCR (Fig. S2A,B) and western blot analysis (Fig. 8A). As compared to the hRet, ADAM10 mRNA expression levels were significantly higher in all RB cell lines investigated (Fig. S2A,B). ADAM17 expression was likewise significantly elevated in five out of seven RB cell lines. The increase, however, did not reach significant levels in RB355 and Rbl13 cells (Fig. S2B). Western blot analyses confirmed the expression of ADAM10 and ADAM17 on protein level in all RB cell lines investigated (Fig. 8A). The 68 kDa active form of ADAM10 was detectable in RB247 and RB383 cells, whereas the inactive 90 kDa precursor of ADAM10 was detectable in all cell lines analyzed except for Rbl30. The 73 kDa precursor and the 60 kDa active form of ADAM17 were clearly detectable in all cell lines, except for RBL13. An endogenous cleavage of the soluble 200 kDa L1CAM ectodomain is distinct in RB355 and also detectable in WERI‐Rb1 and Y79 cell supernatants (Fig. 8A). In the other RB cell line supernatants, L1CAM ectodomain content was below detection levels. A membrane‐bound 32 kDa C‐terminal L1CAM fragment was, however, clearly detectable in cell pellets of all seven RB cell lines investigated (Fig. 8A). In addition, a significant increase in ADAM 10 and ADAM17 expression was observed in RB patient tumors compared to the hRet (Fig. 8B).

**Fig. 9:**
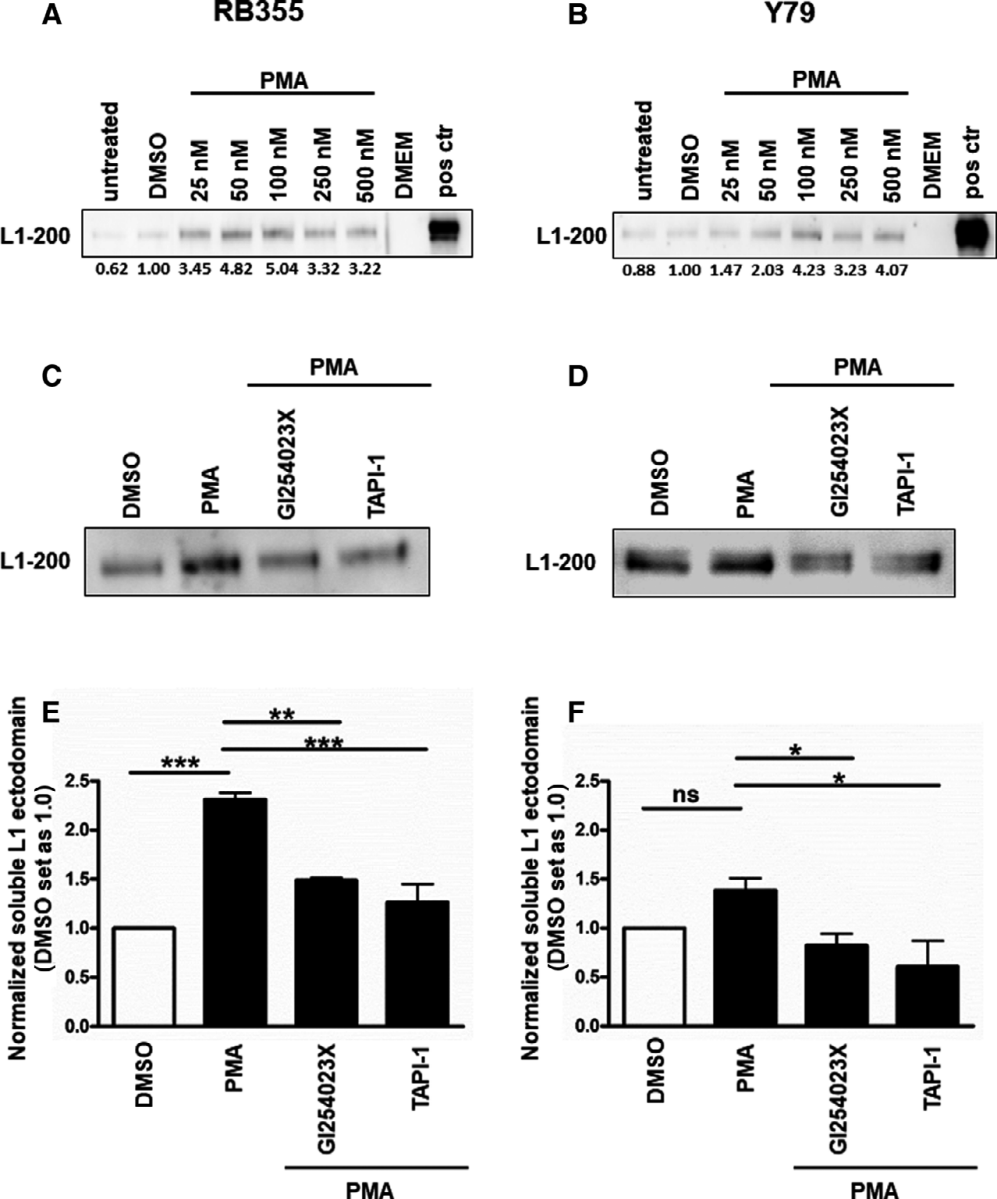
Analysis of ADAM activation by PMA and specific inhibition of ADAM10 by GI254023X and ADAM17 by TAPI‐1 in the RB cell lines RB355 (A, C, E) and Y79 (B, D, F). (A, B) Western blot analysis of ADAM‐mediated L1CAM ectodomain (L1–200) shedding in cell culture supernatant of RB355 (A) and Y79 (B) cells 48h after treatment with different concentrations of PMA. HeLA cell lysate was used as an antibody positive control (pos ctr). DMSO‐treated cells served as a vehicle control and DMEM as a negative control. (C, D) Representative western blots showing activation of L1 shedding upon PMA treatment (50 nm in C; 500 nm in D) and subsequent inhibition of L1 shedding by specific ADAM10 (2 µm GI254023X) and ADAM17 (5 µm TAPI‐1) inhibitors. (E, F) Quantification of L1 ectodomain expression reveals a significant activation of L1 shedding by PMA treatment (50 nm in e; 500 nm in f) and its significant inhibition by administration of ADAM10 (2 µm GI254023X) and ADAM17 (5 µm TAPI‐1) inhibitors in the RB cell lines RB355 and Y79. Values are means of three independent experiments ± SEM. ns *P* > 0.05; *P < 0.05; **P < 0.01; and ***P < 0.001 statistical differences compared to the control group calculated by one‐way ANOVA with Newman–Keuls post‐test.

In order to verify L1CAM shedding by ADAM10 and ADAM17 in the two RB cell lines RB355 and Y79, we activated these ADAMs with increasing concentrations of PMA and could show elevated levels of soluble L1CAM ectodomain in cell supernatants (Fig. 9A,B). Interestingly, in RB355 cells low PMA concentrations (25 and 50 nm; Fig. 9A) already raised L1CAM ectodomain levels in cell supernatants, indicating that this adherent RB cell line seems to be more susceptible for PMA stimulated L1 shedding by ADAMs than Y79 suspension cells (Fig. 9B). PMA stimulation followed by inhibition of ADAM10 (by the inhibitor GI254023X) or ADAM17 (by TAPI‐1) leads to a significant downregulation of L1CAMs ectodomain levels (Fig. 9C–F). The effects of the ADAM inhibitors are likewise higher in adherent RB355 cells compared to Y79 suspension cells (Fig. 9E,F). These data show that both, ADAM 10 und ADAM17, process the soluble L1CAM ectodomain in RB cell lines.

### 3.8 MiRNAs involved in the regulation of L1CAM expression

As we were interested in mechanisms regulating the expression of L1CAM in RBs, we first analyzed the expression pattern of known miRNAs up‐ or downregulating L1CAM in seven RB cell lines. Compared to the hRet, we observed a significant downregulation of miR‐146a‐5p in all RB cell lines investigated (Fig. S3A). MiR‐29‐3p expression is likewise downregulated in all RB cell lines, except for Rbl30 (Fig. S3B), while equal expression levels of miR‐21‐3p, another positive regulator/inductor of L1CAM, were detected when comparing RB cell lines with hRet samples (Fig. S3C).

**Fig. 10:**
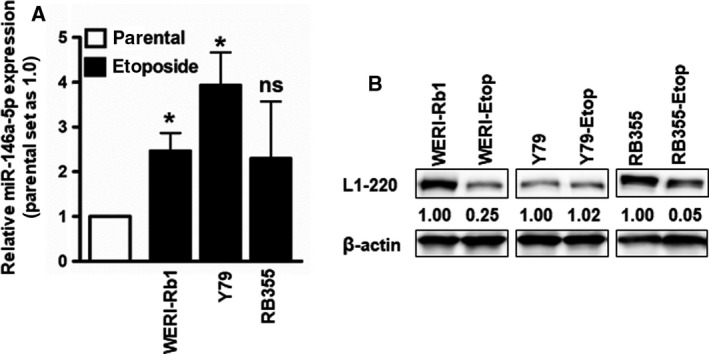
MiRNA‐146a‐5p and L1CAM expression levels in parental, chemosensitive, and etoposide‐resistant WERI‐Rb1, Y79, and RB355 RB cell lines as revealed by real‐time PCR (A) and western blot analysis (B). The indicated intensity ratios relative to ß‐actin, used as a loading control, were calculated using micro manager 1.4 software. Values are means of five independent experiments ± SEM. ns *P* > 0.05 and *P < 0.05 statistical differences compared to the control group calculated by paired Student's *t*‐test.

Next, we were interested in identifying mechanisms regulating L1CAM expression in etoposide‐resistant RB cell lines. We therefore analyzed the expression pattern of miR‐146a‐5p in etoposide‐resistant RB cells in comparison with their parental counterparts. We could show significantly upregulated miR‐146a‐5p expression levels in two out of three etoposide‐resistant RB cell lines investigated (Fig. 10A). Of notion, compared to the parental counterparts, L1CAM protein expression was concordantly downregulated in two out of three etoposide‐resistant RB cell lines investigated (Fig. 10B).

**Fig. 11:**
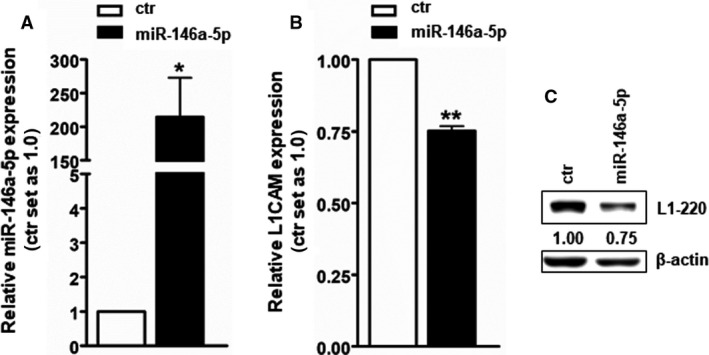
MiR‐146a‐5p overexpression in WERI‐Rb1 cells as revealed by real‐time PCR (A) leads to downregulation of L1CAM protein expression compared to control cells (ctr) as revealed by western blot quantification (B). Indicated western blot lane intensity ratios relative to ß‐actin, used as a loading control, were calculated using micro manager 1.4 software (C). Values are means of three independent experiments ± SEM. *P < 0.05 and **P < 0.01 statistical differences compared to the control group calculated by paired Student's *t*‐test.

In order to prove whether a direct regulation of miR‐146a‐5p leads to an endogenous regulation of L1CAM in RB cells, we exemplarily overexpressed this miR in chemosensitive WERI‐Rb1 cells. We were able to show that L1CAM expression is downregulated after miR‐146a‐5p overexpression in WERI‐Rb1 (Fig. 11), suggesting a miR‐146a‐5p mediated regulation of L1CAM at least in this RB cell line.

In addition, we identified an increased expression of miR‐346 in mainly all RB cell lines compared to the hRet (Fig. S4A) as well as an upregulated expression in etoposide‐resistant RB cells compared to the parental counterparts (Fig. S4B). *In silico* binding analysis identified miR‐346 as a potentially new L1CAM regulating miRNA (Fig. S4C), but the potential binding could not be verified by luciferase assays (Fig. S4D).

### 3.9 L1CAM regulated genes

**Fig. 12:**
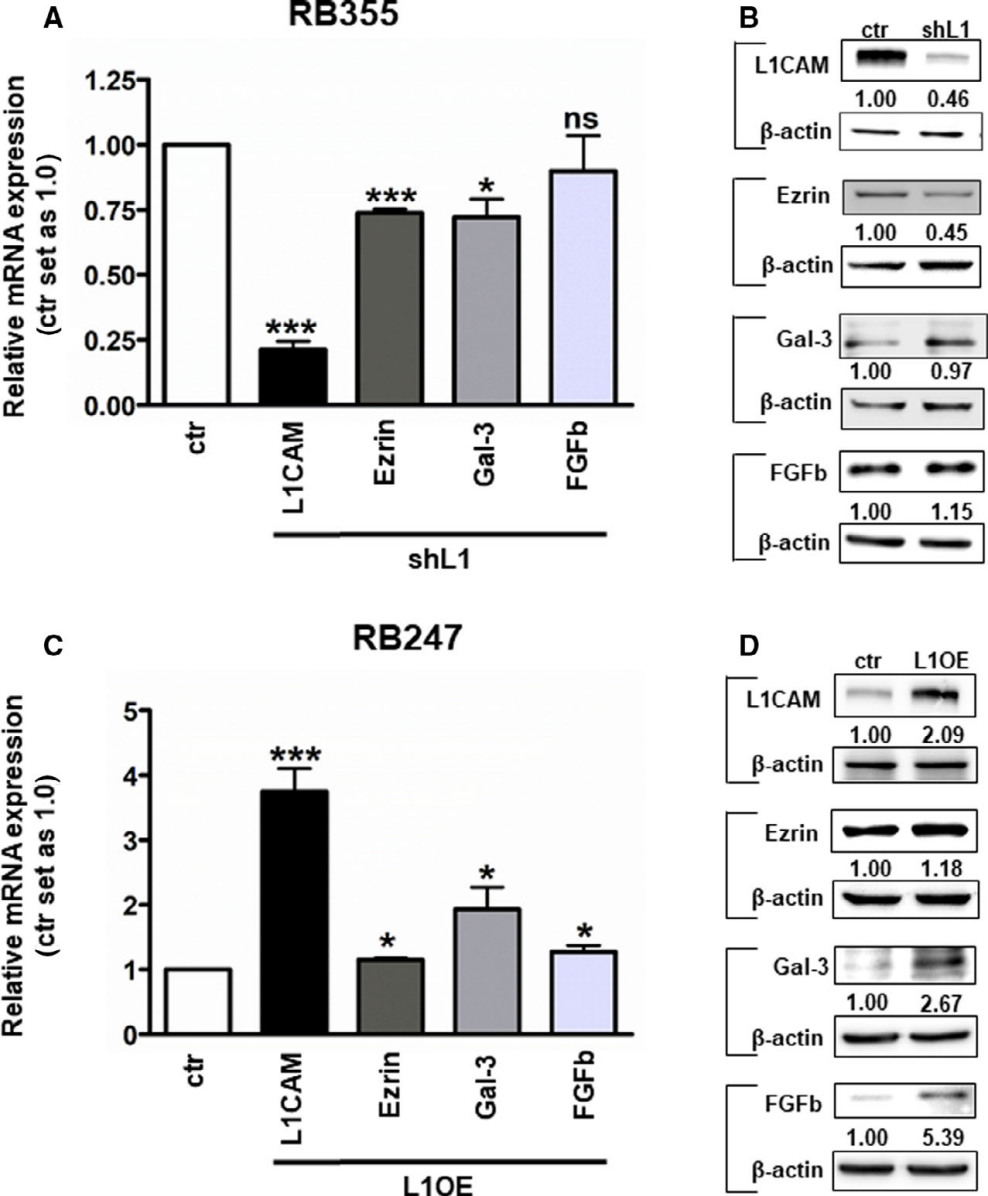
Ezrin, galectin‐3 (Gal‐3), and FGFb expression levels after shRNA‐mediated L1CAM knockdown (shL1) in the RB cell line RB355 (A, B) and stable L1CAM overexpression (L1OE) in RB247 cells (C, D) as revealed by real‐time PCR (A, C) and western blot analysis (B, D). The indicated intensity ratios relative to ß‐actin, used as a loading control, were calculated using micro manager 1.4 software. Values are means of three independent experiments ± SEM. ns *P* > 0.05; *P < 0.05; and ***P < 0.001 statistical differences compared to the control group calculated by paired Student's *t*‐test.

Finally, we set out to identify L1CAM target genes and investigated the expression of ezrin, galectin‐3, and FGFb, which had already been described as L1CAM targets or binding partners in other cancer entities [5, 59]. Except for FGFb, we found mRNA levels of all genes investigated to be significantly downregulated after shRNA‐mediated L1CAM knockdown (Fig. 12A) and ezrin, galectin‐3, and FGFb levels significantly upregulated following stable L1CAM overexpression (Fig. 12C). These changes in expression levels were confirmed on protein level by western blot analysis (Fig. 12B,D), indicating that ezrin, galectin‐3, and FGFb are target genes in L1CAM signaling in RB cells.

## 4 Discussion

In order to further establish L1CAM as a possible therapeutic target in RB treatment, especially in the context of chemotherapeutic resistances, we investigated L1CAM's function in the development and progression of this eye cancer. The results presented indicate that L1CAM may play an important role in RB progression and has an impact on viability and *in vivo* tumor growth of highly aggressive etoposide‐resistant RB cells. In the study presented, we could show that L1CAM is differentially expressed in RB cells compared to the hRet. We analyzed the RB suspension cell lines WERI‐Rb1, Rbl13, Rbl30, RB247, and RB383 as well as the adherent cell line RB355. Our results are consistent with a study describing a differential expression of L1CAM in two RB cell lines Y79 and SNUOT‐Rb1 [38].

L1CAM is involved in tumor progression of several cancer entities, in which high L1CAM expression is associated with advanced tumor stages, metastases, and poor prognoses [60, 61, 62, 63, 64]. In the study presented, L1CAM depletion in RB cell lines significantly decreased cell viability, proliferation, and colony growth and significantly induced apoptosis levels compared to the control cells. These data could be verified by L1CAM overexpression experiments showing the exact opposite effects. Our results are consistent with a previous study reporting that L1 depletion decreases proliferation in Y79 cells [38]. Interestingly, a former study by our group showed that cell lines with lower endogenous L1CAM expression like Rbl 13 and Rbl30 exhibit comparable characteristics to L1CAM knockdown cells, displaying considerably lower doubling times (mean of 94–97 h) compared to those with higher L1CAM levels like WERI‐Rb1 and RB355 (mean doubling time: 50–66 h; [43]), supporting the role of L1CAM in promoting tumorigenicity. Along this line, L1CAM knockdown in oral squamous cell carcinoma and gastric cancer cells results in a significant decrease in cell proliferation, while overexpression of L1CAM in gastric cancer promotes cell proliferation [61, 65]. Other studies could also show that L1CAM‐overexpressing colon carcinoma cells display higher growth rates [9], while downregulation of L1CAM inhibits proliferation of pancreatic cancer cells [66].

A former study reported that in L1‐depleted Y79 cells, the levels of pro‐apoptotic proteins, cleaved caspase‐3, and cytochrome c were significantly increased, whereas the amounts of anti‐apoptotic proteins were reduced [38]. These findings are in good accordance with our data, showing that apoptosis is induced in RB cells after L1CAM knockdown and reduced after L1CAM overexpression. Along this line, caspase‐3/7 assays revealed a significant increase in caspase activity following L1CAM knockdown and a significant decrease in caspase‐3/7 activity upon L1CAM overexpression (Fig. S5).

L1CAM overexpression has been shown to increase anchorage‐independent growth of SNUOT‐Rb1 cells [38]. Besides, knockdown of L1CAM significantly decreased colony formation of prostate cancer cells [67]. In accordance with these results, in our study presented *L1CAM*‐depleted WERI‐Rb1 and RB355 cells formed significantly smaller colonies compared to their parental chemosensitive counterparts.

Here, we show that *L1CAM*‐depleted RB cells inoculated onto the CAM develop significantly smaller and lower weight tumors *in ovo*. Consistently, L1CAM‐overexpressing SNUOT‐Rb1 formed more mass‐like tumors than control cells *in vivo* [38]. Besides, *L1CAM* depletion suppressed tumor growth of glioma, neuroblastoma and ovarian carcinoma and prostate cancer cells in mice [68], while L1CAM overexpression correlates with progression of these tumor entities [69, 70].

In the study presented, *L1CAM‐*depleted RB cells displayed a significantly lower migration rate compared to control cells in CAM assays *in vivo*. In line with our data, other groups likewise reported on a connection between L1CAM expression and cell invasion, motility and metastases in several neuronal and non‐neuronal cancer types [2, 5, 71]. It has been shown that L1CAM depletion abrogates the metastatic potential of T‐cell lymphoma as well as carcinoma cells, reflected by a reduction in migration and invasion of these cells *in vitro* and decreased formation of metastases *in vivo* [72]. In pancreatic and prostate cancer cells, L1CAM knockdown likewise significantly decreased migration and invasion [66], whereas overexpression of L1CAM augments motility and migration of ovarian carcinoma and gastric cancer cells *in vivo* [70].

Since we observed antitumorigenic effects after L1CAM knockdown in RB cells and additionally demonstrated that L1CAM expression seems to be decreased in patients after chemotherapeutic treatment, we hypothesized an effect of L1CAM depletion on chemotherapy‐resistant RB cells. Treatment with chemotherapeutics is one of the main challenges in cancer therapy as resistant cancer cells potentially acquire a more tumorigenic phenotype. Along this line, our group recently demonstrated that etoposide‐resistant RB cells become more aggressive compared to the chemosensitive cells of origin, displaying higher proliferation rates and increased tumor formation capacities [73]. Therefore, developing new treatment strategies and identifying new adjacent or resensitizing molecules for the treatment of tumor cells are main goals in cancer research. After depletion of L1CAM in etoposide‐resistant RB tumor cells followed by retreatment with etoposide, we could show that all cell lines investigated exhibited decreased viability *in vitro*. In addition, L1CAM depletion lowered the viability of etoposide‐resistant RB cells for this chemotherapeutic drug and significantly decreased their tumor growth *in vivo*. Fittingly, other studies already revealed that radio‐ or chemotherapy resistance is induced by upregulation of L1CAM in neuroblastoma [74] and pancreatic cancer [20, 21]. In addition, an increase of invasiveness in 5‐FU resistant pancreatic adenocarcinoma cell lines was functionally linked to L1CAM expression [75]. Continuative to the results of a study depicting that L1CAM depletion decreases cell viability of Y79 RB cells upon short‐term etoposide treatment, while L1CAM overexpression in SNUOT‐Rb1 cells increases drug resistance [38], in the study presented we could show that L1CAM knockdown likewise lowers viability of long‐term etoposide‐resistant RB cell lines upon etoposide retreatment.

L1CAM blocking antibodies have already been shown to inhibit tumor cell growth *in vitro* [76] and in mouse models [77, 78, 79]. In addition, it has been shown that L1CAM is an effective target for CAR T‐cell therapy in RB *in vitro* [80]. In order to propagate L1CAM as a potential clinically valuable therapeutic target it is essential to understand its domain specific expression and functions to give recommendations, which assay could be used in future RB therapies. Thus, we further investigated cleavage of L1CAM by the sheddases ADAM10 and ADAM17. Stimulation with PMA leads to increased soluble L1CAM ectodomain levels in RB cell culture supernatants, whereby subsequent inhibition of ADAM10 or ADAM17 reversed the effect indicating that both ADAMs are involved in ectodomain shedding of L1CAM in RB cells. Our results are in good accordance with data of a study in HEK293T cells, in which PMA treatment increased L1CAM shedding resulting in higher L1‐200 levels in cell culture supernatant of L1CAM and ADAM 10 co‐expressing cells [81]. It has been shown that increased levels of soluble L1CAM ectodomain are induced by an upregulation of ADAM10 in glioma, ovarian cancer, and colon cancer [62, 82, 83]. In general, the soluble L1CAM ectodomain stimulates migration and invasion in other cancer entities [5]. Fittingly, ADAM17 activation is involved in the development of lung [84, 85] and colorectal [86] cancer [87]. Therefore, the implication of ADAM10 and ADAM17 mediating cell migration, protection from apoptosis, and stimulation of cell survival should be further investigated for RB.

It has been shown that both full‐length L1CAM and cytosolic domain of L1CAM interact with ezrin, a cytoskeleton linker protein of the ezrin–radixin–moesin family [5]. In the study presented, we could show that ezrin is downregulated upon knockdown of L1CAM and upregulated following overexpression of L1CAM. In analogy to the effects of our L1CAM knockdown experiments, resulting in downregulation of ezrin expression accompanied by increased apoptosis and decreased proliferation levels, others could show that upregulation of miR‐183‐5p promotes apoptosis and inhibits proliferation, invasion, and migration of human endometrial cancer cells by downregulating ezrin [88]. Additionally, the proproliferative effects seen after L1CAM overexpression in our study could possibly be induced through the upregulation of ezrin, as in colorectal cancer, ezrin also mediates proliferation, motility, and metastatic capacity via L1CAM [5]. Besides, ezrin binding to L1CAM which is elevated in invasive colorectal tumor fronts is essential for proliferation, invasion, and metastasis of colorectal, but also breast and pancreatic cancer cells (for review, see Ref. [5]). In esophageal squamous carcinoma cells, L1CAM upregulates ezrin expression by activating the integrin β1/MAPK/ERK/AP1 signaling pathway resulting in a malignant phenotype [89]. Further along this line, siRNA‐mediated downregulation of ezrin inhibits osteosarcoma cell proliferation, while ezrin overexpression induces proliferation in this tumor entity [90]. Together, these data strongly support the notion that ezrin is a L1CAM downstream target in RB cells.

Studies revealed that the β‐galactoside‐binding protein galectin‐3 (Gal‐3) interacts with L1CAM [59, 91, 92]. In oral squamous carcinoma cells, the inhibition of Gal‐3 significantly reduces the proliferation and invasion and induces apoptosis in this type of cancer cells [93]. Further along this line, inhibition of Gal‐3 expression in human breast cancer cells significantly reduced cell growth rates, anchorage‐independent growth, and thereby colony formation capacity as well as tumor growth in nude mice [94], effects we likewise observed following L1CAM knockdown and concomitant downregulation of galctin‐3 expression in our RB cell lines. Fittingly, Gal‐3 has been demonstrated to promote proliferation and migration and inhibit apoptosis of pituitary tumor cells [95] reflecting the effect of L1CAM overexpression in RB cells, resulting in increased Gal‐3 levels. Thus, it is more than likely that galectin‐3 is involved L1CAM signaling in RB cells.

A study by Mohanan *et al*. [96] demonstrated that the soluble L1 ectodomain acts on glioma cells via FGF receptors (FGFRs) and that L1CAM stimulates glioma cell motility and proliferation via these receptors. Besides, a crosstalk between L1CAM and FGFR signaling was found in epithelial ovarian carcinoma cells [97]. The FGFR ligand FGF2, also known as FGFb, is frequently dysregulated in a variety of cancers (for review, see Ref. [98]) and induces proliferation in WERI‐Rb1 and Y79 RB cells [99]. In the study presented, we found FGFb to be significantly upregulated after L1CAM overexpression fitting the notion that exogenous FGFb induces proliferation and migration and mediates apoptosis prevention [100, 101]. In RB cells, we showed that these exact processes are likewise induced by L1CAM overexpression, indicating that FGFb is a downstream target of L1CAM signaling in RB.

MicroRNAs are mediating post‐transcriptional regulatory processes of gene expression, therefore controlling tumorigenesis and cancer metastasis [102, 103]. Overexpression of oncogenic miRs and downregulation of tumor suppressive miRs play important roles in cancer progression. In the study presented, we analyzed known L1CAM regulating miRs in order to unravel their function in RB tumor progression and chemotherapy resistances. The dysregulation of miR‐146a‐5p is involved in the progression of various cancer entities [102, 104, 105, 106, 107, 108, 109, 110]. Hou *et al*. [111] identified L1CAM as a miR‐146a‐5p target in gastric cancer suppressing the metastatic potential of gastric cancer cells. MiR‐146a is downregulated in gastric cancer and is associated with increased tumor size and poor prognosis [111]. It has likewise been described as a metastasis‐suppressor in breast and pancreatic cancer [104, 108, 109] and decreased miR‐146a expression is correlated with lymph node metastasis and venous invasion in gastric cancer [112, 113]. Of notion, it has already been shown that miR‐146a expression is significantly in RB patient tumors. Besides, miR‐146‐a not only inhibits viability, cell proliferation and invasion, but also increases apoptosis of WERI‐Rb1 and Y79 RB cell lines, suggesting that miR‐146a acts as a tumor suppressor in RB [114]. In the study presented, we could show that miR‐146a‐5p and L1CAM are expressed contrarily in etoposide‐resistant RB cell lines. Moreover, upon miR‐146a‐5p overexpression L1CAM expression is downregulated in WERI‐Rb1 cells, suggesting that at least in some RB cells L1CAM is regulated by this miR.

In summary, targeted therapy to L1CAM or downstream signaling molecules in L1CAM triggered pathways potentially provide promising new treatment options for RBs in general and chemoresistant RB tumors in particular.

## 5 Conclusions

L1CAM expression induces protumorigenic effects in RB, is processed by ADAM10 and ADAM17, and leads to downstream regulation of ezrin, Gal‐3, and FGFb. In addition, L1CAM knockdown results in reduced tumorigenicity and decreases viability and tumor growth of etoposide‐resistant RB cell lines. Therefore, L1CAM and its downstream signaling molecules potentially provide promising new options for targeted therapy of RBs in general and chemoresistant RB tumors in particular.

## Conflict of interest

The authors declare no conflict of interest.

## Author contributions

MB and ND conceptualized the study. MB and OD performed the methodology. MB and OD involved in investigation. KM provided the resources. MB and ND curated the data. ND, MB, and OD wrote the original draft preparation. MB and ND wrote, reviewed, and edited the manuscript. OD visualized the data. ND and MB supervised the study. ND involved in project administration. All authors have read and agreed to the published version of the manuscript. Open Access funding enabled and organized by Projekt DEAL.

## Institutional review board statement

The study was conducted according to the guidelines of the Declaration of Helsinki. The Ethics Committee of the Medical Faculty of the University of Duisburg‐Essen approved the use of human retina (approval #06‐30214) and RB samples (approval #14‐5836‐BO) for research conducted in the course of the study presented.

## Informed consent statement

Informed consent was obtained from all subjects involved in the study.

## Supporting information


**Fig. S1**. Verification of L1CAM knockdown and overexpression as revealed by real‐time PCR analysis.

**Fig. S2**. Quantification of ADAM10 and ADAM17 expression in RB cells compared to hRet as revealed by real‐time PCR analysis.

**Fig. S3**. Quantification of miR‐146a‐5p, miR‐29a‐3p and miR‐21‐3p in different RB cell lines compared to hRet.

**Fig. S4**. Quantification of miR‐346 expression and analysis of miR‐346 binding to the 3′ UTR of L1CAM. (a) Quantification of miR‐346 expression in RB cells compared to healthy retina (hRet) revealed by real‐time PCR indicating a differentially expression of miR‐346 in the cell lines investigated.

**Fig. S5**. Quantification of caspase‐3/7 activity after L1CAM knockdown (shL1) and L1CAM overexpression (L1OE).

## Data Availability

The data that support the findings of this study are available in the supplementary material of this article.
