# Supplementary figures and images for: Role of L1CAM in retinoblastoma tumorigenesis: identification of novel therapeutic targets
Source: Mol Oncol. 2021 Jul 18;16(4):957–81. doi: 10.1002/1878-0261.13054 (PMC8847994; doi:10.1002/1878-0261.13054)

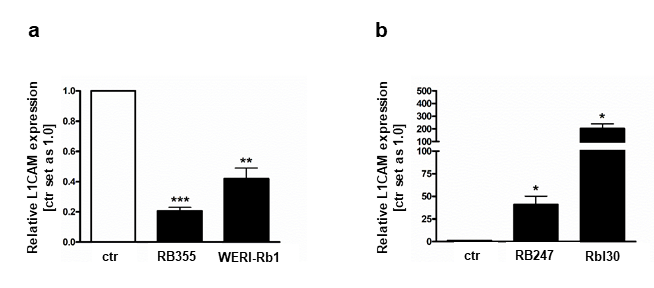

Supplement: Supplementary file 1 — Fig. S1. Verification of L1CAM knockdown and overexpression as revealed by real‐time PCR analysis. [file MOL2-16-957-s005.tif]

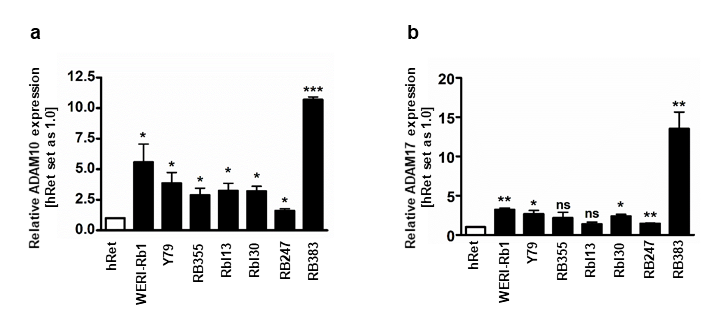

Supplement: Supplementary file 2 — Fig. S2. Quantification of ADAM10 and ADAM17 expression in RB cells compared to hRet as revealed by real‐time PCR analysis. [file MOL2-16-957-s001.tif]

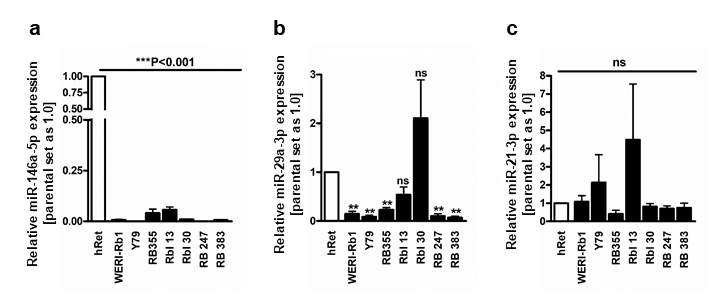

Supplement: Supplementary file 3 — Fig. S3. Quantification of miR‐146a‐5p, miR‐29a‐3p and miR‐21‐3p in different RB cell lines compared to hRet. [file MOL2-16-957-s004.tif]

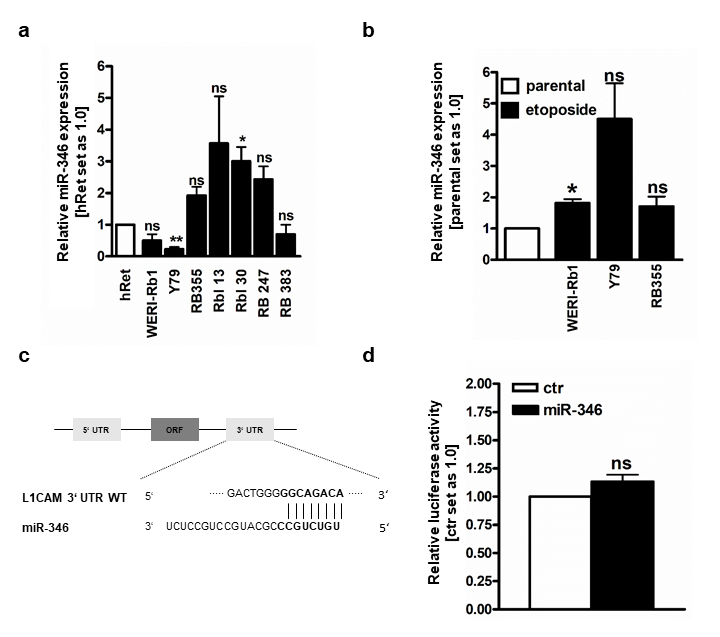

Supplement: Supplementary file 4 — Fig. S4. Quantification of miR‐346 expression and analysis of miR‐346 binding to the 3′ UTR of L1CAM. (a) Quantification of miR‐346 expression in RB cells compared to healthy retina (hRet) revealed by real‐time PCR indicating a differentially expression of miR‐346 in the cell lines investigated. [file MOL2-16-957-s002.tif]

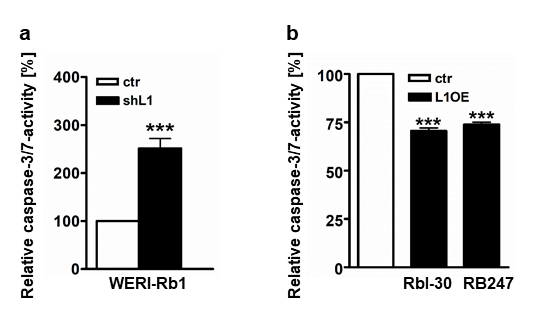

Supplement: Supplementary file 5 — Fig. S5. Quantification of caspase‐3/7 activity after L1CAM knockdown (shL1) and L1CAM overexpression (L1OE). [file MOL2-16-957-s003.tif]
